# Editorial - Our New Board

**DOI:** 10.1177/09544119211054233

**Published:** 2021-10-10

**Authors:** Liz Tanner

## Editorial from Liz Tanner for November/December 2021

As I write this Editorial at the end of September with the new academic year about to start, I realised that I have done nearly 3 months as Editor in Chief of Engineering in Medicine. It has been an interesting, stimulating and educational time. I would like to bring you, our readers, up to date with the changes.

We have renewed the Editorial Board. Three people have ceased to be Associate Editors, Klaus Radermacher and Georges Limbert have decided to step down, but will remain members of the Editorial Board and I will cease to act as an Associate Editor. I would like to thank both Klaus and Georges for their efforts over many years working for Engineering in Medicine, many papers have benefitted from their thoughts and input. I certainly appreciate the time they have put into the journal even if it is not so obvious to the Readers. We have now recruited eight new Associate Editors and you can see them listed in the front cover of the journal and on the journal web page. We previously had only one Associate Editor from China and the Far East and we have recruited Duanduan Chen from Beijing, Eijiro Maeda from Nagoya, Japan and Dalton Tay from Singapore to spread our influence to the East. In the UK we have recruited Damien Lacroix and Koushik Maharatna to broaden our expertises including modelling Artificial Intelligence and Machine Learning. We have recruited Magnús Gíslason from Iceland and Heidi Ploeg and Carolyn Sparrey from Canada going westward again bringing in new expertises. We now have two more women as Associate Editors and I hope that you will consider we have increased our ethnic diversity. I also hope that by reducing the workload on all the Associate Editors we have enabled them to process papers more effectively.

On the Board we have had six retirements, John Fisher, Garth Johnson, David Mitton, Robert Reiner, Antonio Strozzi and Mark Taylor. They have been on the Editorial Board for many years each and we will miss their wise council in meetings. We have our first ever members from Africa and the Middle East and I’m delighted to thank Elsie Kaufmann and Ali Al-Timemy for joining us and expanding our geographical spread. They are joined by Laoise McNamara and Nicholas Dunne from Ireland, Thomas Schauer from Germany, Elizabeth Clarke from Australia, Cheryl Quenneville from Canada and Deepak Vashishth from USA. Consequently, along with being the only one of the 16 journals of the Proceedings of the Institution of Mechanical Engineers with a female Editor in Chief, a quarter of the whole Editorial Board are women.

**Figure 1.**
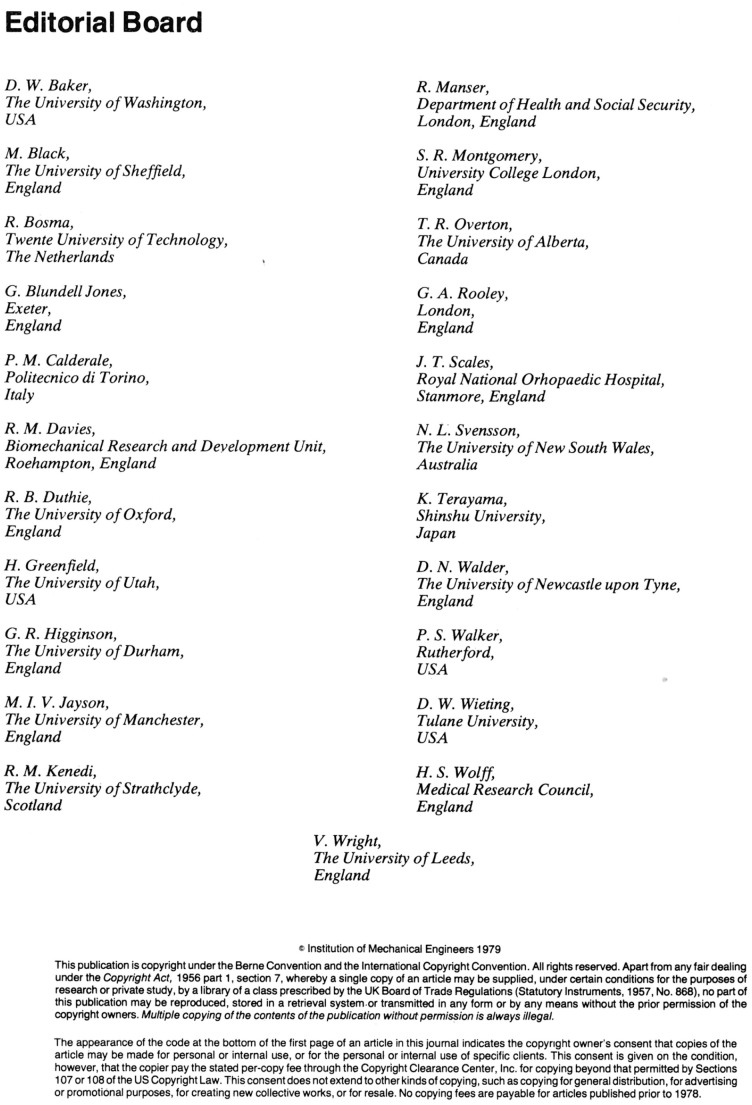
Engineering in Medicine Editorial Board July 1979.


However, I am delighted to point out stability and an extraordinary degree of devotion to Engineering in Medicine, Peter Walker remains on the Editorial Board having been recruited at the start of the journal about 50 years ago, thank you Peter, for a half century of support (Figure 1). I should also say that two of the new recruits reported that, like me, their first published paper was in Engineering in Medicine. We hope that the Editorial Board combines a track record with novel ideas and publishing excellent papers.

We will be looking to recruit further new Board Members next year to ensure that we have the breadth of expertise and geographical spread so that the next half century is one of growth for Engineering in Medicine, however journal publishing changes.

Liz Tanner Queen Mary University of London, London, UK


